# TUSC2(FUS1)-erlotinib Induced Vulnerabilities in Epidermal Growth Factor Receptor(EGFR) Wildtype Non-small Cell Lung Cancer(NSCLC) Targeted by the Repurposed Drug Auranofin

**DOI:** 10.1038/srep35741

**Published:** 2016-11-15

**Authors:** Cao Xiaobo, Mourad Majidi, Meng Feng, Ruping Shao, Jing Wang, Yang Zhao, Veerabhadran Baladandayuthapani, Juhee Song, Bingliang Fang, Lin Ji, Reza Mehran, Jack A. Roth

**Affiliations:** 1 Section of Thoracic Molecular Oncology, Department of Thoracic and Cardiovascular Surgery, University of Texas (UT) MD Anderson Cancer Center, Houston, TX, USA; 2 Department of Bioinfomatics and Computational Biology, UT MD Anderson Cancer Center, Houston, TX, USA; 3 Department of Biostatistics, UT MD Anderson Cancer Center, Houston, TX, USA

## Abstract

Expression of the TUSC2/FUS1 tumor suppressor gene in TUSC2 deficient EGFR wildtype lung cancer cells increased sensitivity to erlotinib. Microarray mRNA expression analysis of TUSC2 inducible lung cancer cells treated with erlotinib uncovered defects in the response to oxidative stress suggesting that increasing reactive oxygen species (ROS) would enhance therapeutic efficacy. Addition of the thioredoxin reductase 1 inhibitor (TXNRD1) auranofin (AF) to NSCLC cells treated with combination of TUSC2 forced expression with erlotinib increased tumor cell apoptosis and inhibited colony formation. TXNRD1 overexpression rescued tumors from AF-TUSC2-erlotinib induced apoptosis. Neutralizing ROS with nordihydroguaiaretic acid (NDGA) abrogated cell death induced by AF-TUSC2-erlotinib, indicating a regulatory role for ROS in the efficacy of the three drug combination. Isobologram-based statistical analysis of this combination demonstrated superior synergism, compared with each individual treatment at lower concentrations. In NSCLC tumor xenografts, tumor growth was markedly inhibited and animal survival was prolonged over controls by AF-TUSC2-erlotinib. Microarray mRNA expression analysis uncovered oxidative stress and DNA damage gene signatures significantly upregulated by AF-TUSC2-erlotinib compared to TUSC2-erlotinib. Pathway analysis showed the highest positive z-score for the NRF2-mediated oxidative stress response. Taken together these findings show that the combination of TUSC2-erlotinib induces additional novel vulnerabilities that can be targeted with AF.

Effective systemic delivery of tumor suppressor genes would have broad applicability in cancer therapeutics as tumor suppressor gene inactivation is the most common genetic abnormality in cancer. TUSC2 (tumor suppressor candidate 2, also known as FUS1) is a tumor-suppressor gene identified in the human chromosome 3p21.3 region, in which allele losses and genetic alterations occur early and frequently in many human cancers, including breast and lung^1^,^2^,^3^. Loss or reduction of TUSC2 expression has been detected in 100% of small-cell lung cancer and 82% of non-small cell lung cancer (NSCLC) cases. Restoration of TUSC2 expression significantly inhibits tumor growth and progression in mouse models^4^. These findings led to a Phase I clinical trial that showed safety and antitumor activity of TUSC2 nanovesicle-based systemic gene therapy administered intravenously in lung cancer patients^5^. TUSC2 nanovesicles combined with erlotinib, an inhibitor of activated epidermal growth factor (EGFR), synergistically inhibited tumor growth and metastases in NSCLC cells with wildtype EGFR by abrogating resistance pathways related to FGFR2 and mTOR activation^6^. We hypothesized that this combination would induce novel vulnerabilities in the cancer cell. One possibility was induction of vulnerabilities related to oxidative stress. A previous study of TUSC2 knock-out mice showed that TUSC2 is involved in mitochondrial function and plays a significant role in mediating stress-induced mitochondrial reactive oxygen species (ROS) in response to chronic oxidative stress^7^. An increase in ROS is associated with abnormal cancer cell growth and reflects a disruption of redox homeostasis due either to an elevation of ROS production or to a decline of ROS-scavenging capacity^8^,^9^,^10^.

Maintaining ROS homeostasis is critical for normal cell growth and survival. Cells control ROS levels by balancing ROS generation and elimination through tightly regulated ROS-scavenging systems. This includes the essential cellular antioxidant thioredoxin, a thiol-dependent electron donor system^11^. In addition to its role in DNA synthesis, thioredoxin directly catalyzes reduction of protein disulfides and regulates the cellular redox environment in a wide range of cellular activities^12^. Thioredoxin reductase 1 (TXNRD1) an NADPH-dependent oxidoreductase enzyme, best known for recycling thioredoxin to its reduced form, is overexpressed in a variety of human cancer cell lines and primary tumors, indicating its tumorigenic involvement^13^. Attention has indeed recently focused on developing specific inhibitors that target TXNRD1^14^. Previous mechanistic characterization has shown that auranofin (AF), an oral, FDA-approved, lipophilic gold-containing compound prescribed for arthritis, inhibits thioredoxin reductase (TXNRD)^15^,^16^. AF interacts with the redox-active site of TXNRD thus preventing electron transfer.

Previously, we have reported a synthetic lethal interaction between the AKT pathway and TXNRD1 via the KEAP1/NRF2 antioxidant system. AF inhibits expression and phosphorylation of major effectors of the PI3K/AKT/mTOR pathway^17^,^18^. In this study, our data support a novel conceptual model in which we identify vulnerabilities in wild type EGFR NSCLC cells following treatment with the TUSC2-erlotinib combination which we found could be targeted with the repurposed drug AF.

## Results

### Elevated ROS level after TUSC2 and erlotinib treatment could be lethal for lung cancer cells

We recently reported that the TUSC2 gene delivered by nanovesicles combined with erlotinib inhibits lung cancer cell viability synergistically^6^. Array analysis was performed to identify potential vulnerabilities in lung cancer cells treated with a combination of TUSC2 nanovesicles and erlotinib. Lung cancer cell lines H157 and H1299 with doxycycline dose response inducible TUSC2 expression were developed using the Tet-On system. Measuring TUSC2 protein expression with various doses of Doxycycline treated cancer cells by western blotting indicated that TUSC2 proteins significantly increased upon Doxycycline treatments whilst β-actin remains unchanged. The TUSC2 expression was positively correlated to Doxycycline doses, shown in supplemental Fig. 1. Microarray mRNA expression analysis of TUSC2 inducible lung cancer cells treated with erlotinib, illustrated in Supplemental Figure 2A,B, uncovered defects in the response to oxidative stress including downregulation of HSPA6, IFNL2, PPP1R15A (GADD34), and GADD45B. This result suggested that increasing ROS would be lethal for the cancer cells thus enhancing therapeutic efficacy.

### AF-TUSC2-erlotinib enhance tumor cell death synergistically

**Figure 1:**
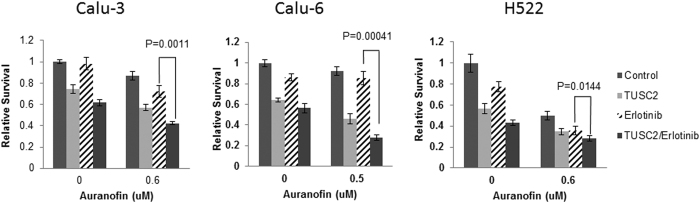
AF-TUSC2-erlotinib enhance tumor cell death significantly. Wild type EGFR NSCLC Calu-3, Calu-6, and H522 were transiently transfected with 4 μg TUSC2 cDNA, treated with 0.5–0.6 μM auranofin and/or 1 μM erlotinib and assayed for viability using XTT assay. Data represent the mean of three independent experiments each in quadruplicates, and was statistically analyzed using a three-way ANOVA and two-tailed t test. After adjusting for multiple comparisons, a significant inhibitory effect was found for the TUSC2-erlotinib-AF combination compared to any other treatment. Data shown represent the mean ± SE of three independent experiments.

We hypothesized that inhibition of TXNRD1 by AF should increase cellular ROS thus increasing lethality in the presence of TUSC2-erlotinib. We next tested the effect of AF on cells treated with TUSC2-erlotinib. Cell viability was evaluated in three wild type EGFR TUSC2-deficient NSCLC cell lines, Calu-3, Calu-6 and H522. Cells were transiently transfected with TUSC2 cDNA and treated with clinically achievable concentrations of AF and erlotinib. Treatment groups were: (1) 0.5–0.6 μM AF; (2) 1 μM erlotinib; (3) 0.5–0.6 μM AF plus 1 μM erlotinib. Untreated cells and cells treated with AF, erlotinib, or AF and erlotinib were used as controls. AF alone had a minimum inhibitory effect on Calu-3 and Calu-6, but reduced viability of H522 by 50% (Fig. 1). In all three cell lines, TUSC2 forced expression reduced viability more effectively than either AF or erlotinib. TUSC2 transient expression combined with AF and erlotinib reduced viability of Calu-6, Calu-3, and H522 by 78%, 64%, and 75% respectively, which was more effective than any other tested treatments.

**Figure 2:**
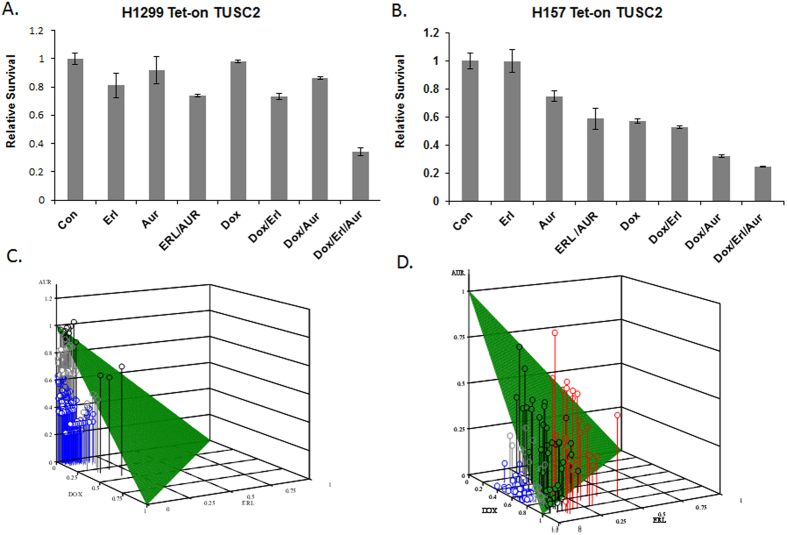
Synergistic effect of AF-TUSC2-erlotinib combination. (**A,B**) Wild type EGFR TUSC2 Tet-On stable clones H157 and H1299 were treated with 1 μg/ml doxycycline (TUSC2 expression); 2.3 μM erlotinib; 0.5 μM auranofin; 1 μg/ml doxycycline/2.3 μM erlotinib; 1 μg/ml doxycycline/0.5 μM Auranofin; 2.3 μM erlotinib/0.5 μM Auranofin; and 1 μg/ml doxycycline/1 μg/ml doxycycline and 0.5 μM auranofin for 72 hours. Untreated cells were used as control. Cell viability was assessed with XTT assay. After adjusting for multiple comparisons, a significant inhibitory effect was found for the TUSC2-erlotinib-AF combination compared to any other treatment using three-way ANOVA and two-tailed t test. Data shown represent the mean ± SE of three independent experiments. (**C,D**) Loewe additivity model was used at 95% confidence intervals (CI); II < 1, II = 1, and II > 1 correspond to the drug interactions’ being synergistic, additive, and antagonistic, respectively. Blue needles represent synergy; Grey represents combination of the three drugs that had interaction index (II) less than one without synergy; Black represents combination of the three drugs with an interaction index (II) above one without antagonism; Red and green surface represent antagonistic and additive effects respectively.

Next, we evaluated the effect of TUSC2-erlotinib-AF combination on two wildtype EGFR Tet -inducible TUSC2 NSCLC cell clones H157 and H1299. Doxycycline treatment was used to induce TUSC2 expression which was confirmed by western blot in every experiment. Consistent with the above results, cell death by TUSC2-erlotinib-AF was higher than TUSC2-AF, TUSC2-erlotinib, or AF-erlotinib. Viability of H1299 and H157 was reduced by over 70% and 80% respectively (Fig. 2A,B). Turkey’s multiple comparisons test was performed for pairwise differences between different treatment groups and found that TUSC2-erlotinib-AF had the highest probability of a cooperative effect (>98%).

To further characterize the interaction between AF, TUSC2, and erlotinib, and determine its magnitude, we assessed viability at different individual dose-levels. Five cell viability measurements were taken at each different combination: Doxycycline (0, 0.4, 0.6, 0.8, 1.0, and μg/ml), Erlotinib (0, 0.6, 0.9, 1.2, 1.8, and 2.3 μM), and AF (0, 0.5, 0.75, 1, 2, and 3 μM). After subtracting background, measurements were divided by average viability at AF = 0, doxycycline = 0, and erlotinib = 0. We used the Loewe additivity model at 95% confidence intervals. Isobolograms was used to generate Interaction Index (II). II < 1, II = 1, and II >1 correspond to the drug interactions’ being synergistic, additive, and antagonistic, respectively. For the H1299 clone, the optimum synergy was observed at 0.25 μg/ml doxycycline, 0.5 μM erlotinib, and 0.5 μM AF; 0.25 μg/ml doxycycline, 0.75 μM erlotinib, and 0.5 μM AF; 0.5 μg/ml doxycycline, 0.75 μM erlotinib, and 0.5 μM AF; 0.75 μg/ml doxycycline, 0.5 μM erlotinib, and 0.75 μM AF. Similarly, for the H157 clone, optimum synergy was observed at 1 μg/ml doxycycline, 0.6 μM erlotinib, 0.5 μM AF; and at 1.2 μg/ml doxycycline, 0.6 μM erlotinib, and 0.5 μM AF. Synergy was also observed at auranofin and erlotinib concentrations between 1–3 μM. Figure 2C,D shows results represented in isobolograms for H1299 and H157 respectively. These results indicate that AF-TUSC2-erlotinib combination synergistically induces cell death of wild type EGFR Dox-inducible TUSC2 H1299 and H157 clones at nanomolar concentrations.

### AF-TUSC2-erlotinib reduce tumor cell colony formation synergistically

**Figure 3:**
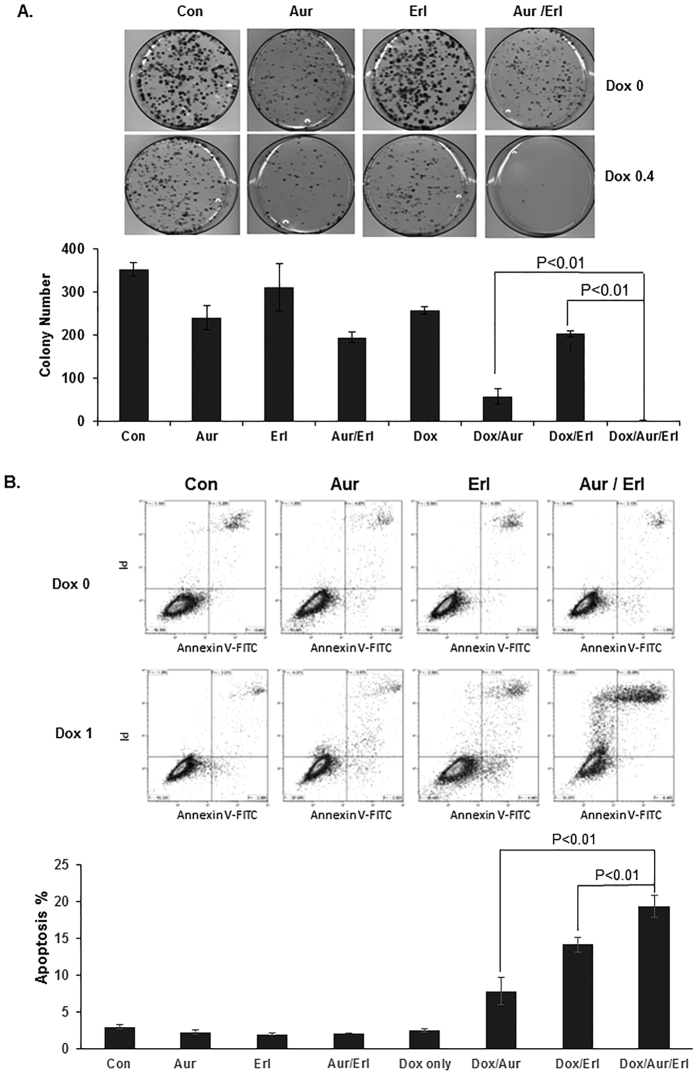
Auranofin/TUSC2/erlotinib reduce tumor cell colony formation and enhance apoptosis synergistically. (**A**) H1299 Tet-inducible TUSC2 clone was grown in RPMI-1640 medium containing 400 μg/ml G418 prior to treatment with: (1) 0.4 μg/ml Doxycycline; (2) 0.2 μM Auranofin; (3) 0.8 μM erlotinib; (4) 0.4 μg/ml Doxycycline/0.2 μM Auranofin; (5) 0.2 μM Auranofin/0.8 μM erlotinib; and (6) 0.4 μg/ml Doxycycline/0.2 μM Auranofin/0.8 μM erlotinib. Survived colonies were fixed with 3.7% formalin, stained with 1% crystal violet, counted with a stereomicroscope, and analyzed with Image-J software. Values represent the mean of three independent experiments. The statistical significance of differences between treatments was calculated by using three-way ANOVA and two-tailed t test; P < 0.05 was considered significant. The cooperative effect of DC-TUSC2 and erlotinib combination was assessed using a Bayesian parametric bootstrapping approach. The Statistical software S-PLUS 8.0 was used for all analyses. (**B**) Following treatment of H1299 Tet-inducible TUSC2 clone with 1 μg/ml Doxycycline, 0.8 μM Auranofin, and 0.8 μM Erlotinib, cells were harvested, washed with PBS, and stained with Annexin-V (Promega, Madison, WI). Apoptotic activity was measured by flow cytometry using FlowJo software (Tree Star Corp, Ashland, OR). Values represent the mean of three independent experiments, each in quadruplicate.

To further evaluate the biological activity of the TUSC2-erlotinib-AF combination, we tested its effect on tumor cell colony formation. In dose titration experiments at nanomolar concentrations, we found that the ability of the wild type EGFR Dox-inducible TUSC2 H1299 clone to form colonies was reduced to almost 100% (Fig. 3A). AF, TUSC2, and erlotinib, reduced colonies by 30%, 25%, and 10%, respectively. AF-erlotinib or TUSC2-erlotinb reduced colonies by 50%, whereas AF-TUSC2 treatment resulted in 80% inhibition. AF-TUSC2-erlotinib inhibited colony formation to the greatest extent (>95%) which is consistent with the observed cell viability results.

### AF-TUSC2-erlotinib induces apoptosis

We analyzed whether the observed synergy between AF, TUSC2, and erlotinib involves apoptosis. AF-TUSC2-erlotinib resulted in a seven fold increase in apoptosis compared to the control which was greater than each individual agent or the two agent combinations (Fig. 3B). These results suggest that apoptosis contributes to the synergistic tumor cell death by AF-TUSC2-erlotinib.

### AF-TUSC2-erlotinib inhibits tumor growth and prolongs survival of xenograft mice

**Figure 4:**
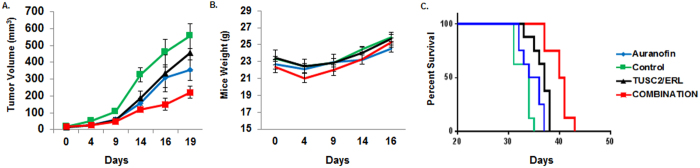
Auranofin/TUSC2/erlotinib inhibits tumor growth and prolong animal survival significantly. A subcutaneous mouse model of the human NSCLC using H1299 cells was adopted to evaluate effect the trinary combination: Auranofin plus systemic delivery of DC-based TUSC2 nanoparticles, and erlotinib. (**A**) Tumor volumes were calculated taking length of the longest diameter across the tumor and with width of the corresponding perpendicular diameter using the formula: length x width^2^ × 0.52. The rate of tumor growth inhibition was calculated as 100% × (treated tumor size/control tumor size). (**B**) Mice were weighed throughout the experiments. (**C**) Overall survival of animals with various treatments were estimated by Kaplan-Meier method. Log-rank (Mantel-Cox) test was performed to compare overall survival between TUSC2/erlotinib treated mice and TUSC2/erlotinib/Auranofin treated mice (P < 0.0001).

To investigate the efficacy of AF-TUSC2-erlotinib *in vivo*, eight wild type EGFR H1299-derived xenograft mice were allocated to each treatment group: DOTAP: cholesterol (DC)-empty vector; AF; DC-TUSC2 nanoparticle complex-erlotinib; and DC-TUSC2-AF-erlotinib. The DC-TUSC2-AF-erlotinib combination inhibited tumor growth more effectively than AF or TUSC2-erlotinib (Fig. 4A). The mean tumor volumes for control, AF, TUSC2-erlotinib, and AF-TUSC2-erlotinib were 600.29 ± 311.17; 353.30 ± 172.28 mm^3^; 456.94 ± 172.28 mm^3^, and 222.03 ± 98.14 mm^3^, respectively (p < 0.005). The posterior probability of a cooperative effect between AF, TUSC2 and erlotinib was greater than 99%, which means that there were less than 1 in 100 chance that the effect of this combination was not cooperative. DC-TUSC2-AF-erlotinib had no apparent effect on weight or change in food or water intake (Fig. 4B). DC-TUSC2-AF-erlotinib prolonged animal survival (median 44 days) compared with AF (median 32 days) or erlotinib/TUSC2 (median 36 days), shown in Fig. 4C.

### TRNXD1 inhibition renders cells more sensitive to TUSC2-erlotinib treatment

**Figure 5:**
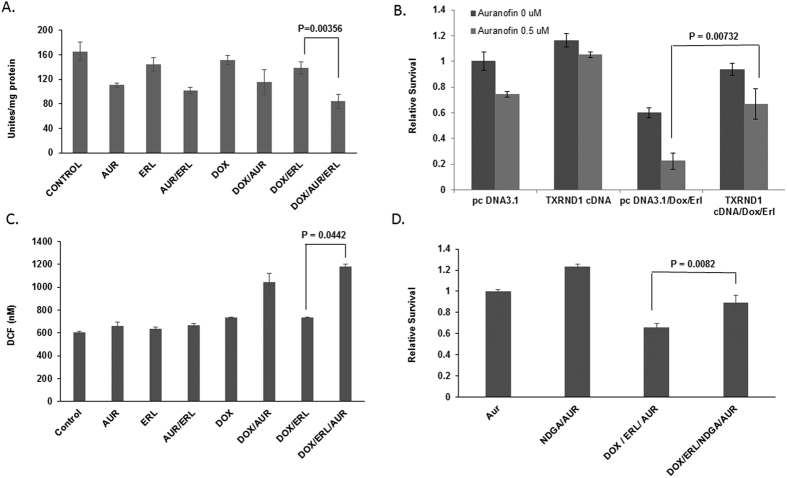
TRNXD1 inhibition renders cells more sensitive to TUSC2 and erlotinib treatment and involves increase in ROS levels. (**A**) Thioredoxin Reductase 1 (TXNRD1) activity was measured using Thioredoxin Reductase 1 (TXNRD1) Activity Assay Kit with TXNRD1 antibody. Following treatments, Tet-inducible TUSC2 H1299cell lysates were incubated in the absence or presence of DTT (10 mM) at 37 °C in TE buffer. TXNRD1 was purified using Chroma Spin TE-10 columns and incubated with increasing concentrations of NAPQI (0.1–100 μM) or DMSO. TXNRD1/insulin mixture (50 nM purified rat liver TXNRD1, 0.5 mM NADPH, and 170 μM insulin in TE buffer) was added and changes in absorbance at 340 nm were recorded. TXNRD1 activity was calculated as the linear change in absorbance per min and expressed as a percentage of the enzyme activity of DMSO-treated control samples. (**B**) H1299 cells were transfected with 1 μg TXNRD1 cDNA and treated as indicated before assayed for survival with XTT assay. (**C**) H1299 Tet-inducible TUSC2 cells were cultured in a black 96-well culture plates and washed with HBSS before incubation in 100 μl of 1X cell-permeable fluorogenic probe 2’, 7’-Dichlorodihydrofluorescin diacetate (DCFH-DA) at 37 °C for 60 minutes. After TUSC2 induction with 1 μg doxycycline for 24 hours, cells were treated with 0.7 μM auranofin and/or 0.9 μM erlotinib for two hours. All treatment were in triplicates. Fluorescence was analyzed with a fluorometric plate reader at 480/530 nm. Hydrogen peroxide, H_2_O_2_ at 1000 μM was used a positive control. DCFH-DA treatment was 60 minutes. (**D**) H1299 cells were treated with 5 μmol/L NDGA before exposure to the indicated treatments and assayed for cell survival with XTT assay. TUSC2 induction by doxycycline was confirmed by western blot in all experiments. Data shown represent the mean ± SE of three independent experiments.

We analyzed the role of TXNRD1, the primary target of auranofin, in the observed AF-TUSC2-erlotinib synergy. TXNRD1 enzymatic activity was measured after exposure of the wild type EGFR Tet-inducible TUSC2 H1299 clone to AF-TUSC2-erlotinib. AF reduced TXNRD1 activity by 35%, which is consistent with previous reports (Fig. 5A). Doxycycline, erlotinib, and doxycycline-erlotinib slightly reduced TXNRD1 enzymatic activity. Doxycycline-AF and AF-erlotinib reduced TXNRD1 to the extent of auranofin alone whereas AF-Dox-Erlotinib treatment resulted in a 50% inhibition. TXNRD1 overexpression abrogated the ability of AF to enhance sensitization to doxycycline-TUSC2 (Fig. 5B). These results suggest that TXNRD1 inhibition renders cells more sensitive to TUSC2-erlotinib.

### AF-TUSC2-erlotinib synergy is mediated through increased ROS production

We have previously reported that AF alters cellular ROS balance^17^. Therefore we analyzed whether AF-TUSC2-erlotinib interaction is regulated by ROS. ROS expression levels were assessed in wild type EGFR Tet-inducible TUSC2 H1299 clone after exposure to the AF-TUSC2-erlotinib combination. AF, erlotinib, doxycycline, AF/erlotinib, and doxycycline-erlotinib had a minimal effect (Fig. 5C). In contrast, compared to control, doxycycline-AF and doxycycline-AF-erlotinib enhanced ROS levels by 80% and almost 100%, respectively. To determine whether ROS is required for synergy by AF-doxycycline-erlotinib, cells we treated with ROS blocker nordihydroguaiaretic acid (NDGA; 5 μM) before exposure to the combination. As Fig. 5D shown, NDGA reversed cell growth inhibition, which indicates that AF-doxycycline-erlotinib efficacy occurs through ROS.

### Gene expression profile and pathway analysis

**Figure 6:**
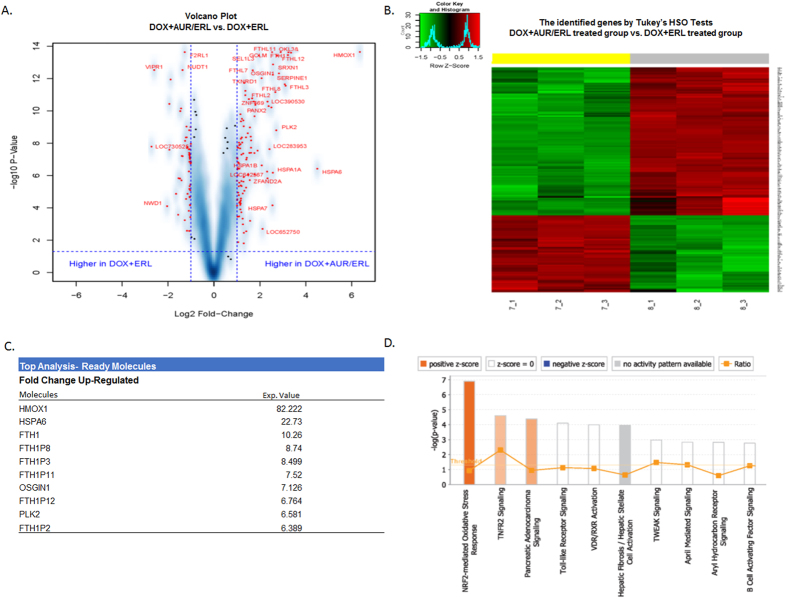
Gene expression profile and pathway analysis. Illumina array Human HT-12V4 expression bead chip platform was used across the Tet-inducible TUSC2 H1299 clone treated with auranofin-doxycycline-erlotinib vs doxycycline-erlotinib. Each treatment group was in triplicates. Beta-Uniform Mixture models were used to adjust for multiple comparisons. Significant genes were identified by One Way ANOVA, and Tukey’s HSD tests were used to analyze pairwise comparisons. Fold change larger than 2 or smaller than −2 were considered as significant at p < 0.05. (**A**) Volcano plot: 164 genes marked in red at FDR of 0.0001 were selected. (**B**) Heatmap: Genes with FDR of 0.0001 and fold change >2 were considered as significant genes. doxycycline-erlotinib: gray, auranofin-doxycycline-erlotinib: yellow. (**C**) List of top genes significantly upregulated between binary and trinary combination treatment. (**D**) Ingenuity pathway analysis Pathway analysis based on statistical significance and strength of association with extracts revealed the highest positive z-score for NRF2-mediated oxidative stress response.

To identify differentially expressed genes and specific pathways involved in AF-TUSC2-erlotinib synergy, we used Illumina Human HT-12V4 expression bead chip platform across the TUSC2-inducible H1299 clone. The dataset for each of the control and seven treatment groups (AF, erlotinib, doxycycline, AF-erlotinib, AF-TUSC2, erlotinib-TUSC2, and AF-TUSC2-erlotinib) were analyzed individually to generate specific sets of genes for which expression levels were up or downregulated between erlotinib-TUSC2 and AF-TUSC2-erlotinib treatments. Volcano plots and Heatmap were used to visualize the results (Fig. 6A,B). The resulting aggregate scores were used for pathway analysis based on the selected genes at a false detection rate (FDR) of 0.0001. The results show a gene profile that differentiates between erlotinib-TUSC2 and AF-TUSC2-erlotinib (Fig. 6B). Genes that are upregulated all encode proteins for oxidative stress and DNA damage (Fig. 6C). Their expression was activated by 6.389 to 82.222 fold with HMOX1, HSPA6 and FTH1 being the highest. Ingenuity pathway analysis showed the highest positive z-score for the NRF2-mediated oxidative stress response pathway (Fig. 6D). These results support an important role for antioxidant and DNA damage response gene networks in AF-TUSC2-erlotinib synergy.

## Discussion

Molecular vulnerabilities in lung cancer are frequently not targetable by existing drugs. We identified a gene delivery strategy that creates vulnerabilities in pathways susceptible to existing drugs. Restoration of the TUSC2/FUS1 tumor suppressor gene, which also functions as a multikinase inhibitor, enhances killing of EGFR wild type human lung cancer cells by the EGFR tyrosine kinase inhibitor erlotinib^4^,^6^,^19^. TUSC2/erlotinib is currently in Phase II clinical trials in stage 4 NSCLC patients who are EGFR wildtype and have tumor progression on other treatments^5^. In this report, we found that addition of thioredoxin reductase 1 (TXNRD1) inhibitor auranofin to the TUSC2/erlotinib combination synergistically enhanced cancer cell death by induction of apoptosis through an increase in ROS. We tested several wild type EGFR NSCLC lines with different histologies and genetic backgrounds to determine whether the observed effects were specific to certain cells or genomic subtypes. These cell lines have low to absent levels of TUSC2 expression. Restoration of TUSC2, both transient and stable, sensitized these cell lines to erlotinib, an effect that was clearly enhanced after AF addition at clinically achievable concentrations. The efficacy of AF-TUSC2-erlotinib *in vitro* was validated *in vivo* in mice bearing NSCLC tumor xenografts.

A quantitative isobologram statistical model was used to characterize the interactions among AF, TUSC2, and erlotinib. We combined dose-response curves for each combination with one, two, and three drugs, and compared multiple concentrations in the nanomolar range. AF, TUSC2, or erlotinib each produced similar tumor cell killing effects, although that of TUSC2 was slightly better. When combined at low doses, efficacy was statistically superior for AF-TUSC2-erlotinib, compared to that of the individual effects indicating a departure from additivity, which indicates synergy.

TXNRD1 is the only enzyme known to catalyze the reduction of thioredoxin and hence is a central component in regulation of reactive oxygen species^11^,^12^. Expression and activity of TXNRD1 has been reported to be upregulated in various tumors, and interconnection between its inactivation and cell growth inhibition and apoptosis has been shown^13^,^14^. AF, alone, or combined with TUSC2-forced expression or erlotinib reduced TRND1 enzymatic activity at different potency levels, however the three agent combination was the most effective. TXNRD1 overexpression rescued cells from AF/TUSC2/erlotinib induced cell death, which indicates that this enzyme plays a functional role in the combined efficacy. Reducing ROS with NDGA abrogated the effect of AF on TUSC2-erlotinib. Thus, it is conceivable that AF improves sensitivity of wild type EGFR NSCLC to TUSC2-erlotinib by mediating increased ROS via inhibition of TXNRD1 in cells rendered less responsive to oxidative stress.

Microarray mRNA expression analysis revealed an oxidative stress and DNA damage gene signature differentially regulated between TUSC2-erlotinib and auranofin-TUSC2-erlotinib combination treatments. Ten genes in encoding proteins of iron and oxygen metabolism were significantly upregulated: HMOX1, HSPA6, FTH1, FTH1P8, FTH1P3, FTH1P11, OSGIN1, FTH1P12, PLK2, and FTH1P2. Pathway analysis showed the highest positive z-score for NRF2-mediated oxidative stress response. Nrf2 is a transcriptional regulator that targets heme oxygenase-1 (HMOX1), ferritin (FTH) genes, oxidative Stress induced growth inhibitor 1 (OSGIN1), and TXRD1. These genes function to protect cells from oxidative damage during stress and are implicated in cancer initiation, progression and inhibition. In NSCLC patients, NRF2 is frequently deregulated and may effect tumor initiation and progresson^19^,^20^. These results are consistent with our recent report of synthetic lethality between the TXNRD1 and AKT pathways which occurred through the KEAP1/NRF2 cellular antioxidant axis^17^. NRF2 controls expression and activity of TXNRD1 and is activated by the ROS and AMP-activated protein kinase (AMPK) signaling system, which has emerged in recent years as a regulator of the redox-state of the cell^21^,^22^. AMPK activation was proposed to sensitize NSCLC cells to erlotinib^23^. It is worth noting that TUSC2 directly activates AMPK allowing AMPK to drive cell dearth more effectively^5^,^24^.

In this paper we show that the combination of TUSC2 and erlotinib induces additional novel vulnerabilities that can be targeted with an approved drug, auranofin. Thus interrogation of genetically modified lung cancer cells may reveal novel vulnerabilities targetable by existing drugs. A clinical trial showed that the TUSC2 gene can be delivered intravenously in patients using nanovesicles and achieves sufficient TUSC2 protein expression levels in tumors to mediate response^5^. Thus the combination of these drugs is feasible in future clinical trials for patients with wild type EGFR.

## Materials and Methods

### Cell Culture

Wild type EGFR human non-small cell lung adenocarcinoma cell lines with low or absent TUSC2 expression, H1299, H522, and H157 were provided by Drs. John V. Heymach (MD Anderson Cancer Center), Adi Gazdar and John D. Minna (The University of Texas Southwestern Medical Center at Dallas). Calu-3 and 6 cells were purchased from ATCC I (Manassas, VA). Cells were maintained in RPMI-1640 medium (Manassas, VA), supplemented with 10% heat-inactivated fetal bovine serum (Invitrogen, Carlsbad, CA).

### Reagents

Erlotinib was obtained from the clinical pharmacy at the University of Texas MD Anderson Cancer Center (MDACC, Houston, TX). Auranofin, NDGA, and Doxycycline, were purchased from Sigma-Aldrich Corporation (Saint Louis, MO). TXNRD1 activity assay kit was purchased from abcam, (Cambridge MA). TUSC2 polyclonal antibody was developed in Bethyl Laboratories, Montgomery, TX. ROS kit was purchased from Cell Biolabs Inc (San Diego, CA). Lipofectamine 2000 was purchased from Invitrogen Corporation (Carlsbad, CA). DOTAP: Cholesterol (DC)–TUSC2 complexes were made as previously described^4^.

### Stable Tet-inducible TUSC2 Cell Lines

Tet-inducible TUSC2 expressing cells were produced using Lenti-X Tet-On advanced inducible expression system (Clontech, Mountain View, CA) according to the manufacturer’s instructions. Briefly, H1299, and H157 cells were infected with lentivirus generated with the pLVX-Tet-On advanced vector to constitutively express the tetracycline-controlled transactivator. After G418 selection, surviving colonies were expanded and infected with pLVX-Tight-Puro-Luc. New colonies were selected and expanded under G418 and puromycin. Surviving clones were selected after exposure to 2 μg/ml doxycycline for 48 hours, and assessed for TUSC2 expression by Western blotting.

### Cell viability assay

We used 2,3-bis(2-methoxy-4-nitro-5-sulfophenly)-5-[(phenylamino) carbonyl]-2H-tetrazolium hydroxide (XTT) assay to evaluate overall treatment effect on cell viability. Calu3, Calu 6, and H522 were transfected with 4 μg TUSC2 cDNA before treatment with 0.8 μM erlotinib and/or 0.5 μM AF. Combined treatments for TUSC2 stable clones H1299 and H157 were: 1 μg/ml doxycycline plus 1 μM erlotinib; 1 μg/ml doxycycline and 0.5 μM AF; 1 μM erlotinib and 0.5 μM AF; and 1 μg/ml doxycycline and 0.5 μM AF and 1 μM erlotinib for 72 hours. Several other concentrations at nanomolar ranges of AF, doxycycline, erlotinib were used to test for synergy. Untreated cells and cells treated with AF, doxycycline, or erlotinib served as controls. The statistical significance of differences between treatments was calculated using three-way ANOVA and two-tailed t test; P < 0.05 was considered significant. The percentage of viable cells was determined by the ratio of absorbance of treatment and control groups: ODT/ODC × 100% from three independent experiments, each in triplicates. Average of three cell viability measurements were calculated after subtracting background and divided by average cell viability at AF = 0, doxycycline = 0, and erlotinib = 0.

### Colony Formation Assay

H1299 Tet-inducible TUSC2 clone was grown in RPMI-1640 medium containing 400 μg/ml G418 prior to treatment with: (1) 0.4 μg/ml doxycycline; (2) 0.2 μM AF; (3) 0.8 μM erlotinib; (4) 0.4 μg/ml doxycycline/0.2 μM AF; (5) 0.2 μM AF/0.8 μM erlotinib; and (6) 0.4 μg/ml doxycycline/0.2 μM AF/0.8 μM erlotinib. Survived colonies were fixed with 3.7% formalin, stained with 1% crystal violet, counted with a stereomicroscope, and analyzed with Image-J software. Values represent the mean of three independent experiments.

### Flow Cytometry/apoptosis assays

Following treatment of Tet-inducible TUSC2 H1299 clone with 1 μg/ml doxycycline, 0.8 μM AF, and 0.8 μM erlotinib, cells were harvested, washed with PBS, and stained with Annexin-V (Promega, Madison, WI). Apoptotic activity was measured by flow cytometry using FlowJo software (Tree Star Corp, Ashland, OR). Values represent the mean of three independent experiments, each in quadruplicate.

### Animal experiments and analysis of tumor growth

Protocols were approved by the Ethics of Animal Experiments committee, The University of Texas MD Anderson Cancer Center, in accordance with NIH Guidelines for the Care and Use of Laboratory Animals. Six to eight week old Balb/c athymic nude mice female were purchased from Harlan Sprague Dawley (Indianapolis, IN). Animals were inoculated subcutaneously with wild type EGFR TUSC2-deficient human H1299 cells (3 × 10^6^). When tumors reached an average volume of 0.1 cm^3^, mice were randomly divided into four treatment groups (n = 8 animals per group): (1) PBS control; (2) AF (10 mg/kg) delivered once daily five times per week via i.p. injection for two weeks; (3) Erlotinib (30 mg/kg) orally feed daily with a total of 8 times, and intravenous injections with DC-TUSC2 complexes at a dose of 25 μg of plasmid DNA, 10 nmol DC in 100 μL of 5% dextrose in water every 48 hours for a total of three injections; and (4) AF-TUSC2-Erlotinib combination. Mice were sacrificed when tumors reached a size of 1.5-cm diameter. Evaluation of tumor size was made without knowledge of the treatment groups. Tumor volumes were analyzed at day 0, 3, 8, 14, 16, and 19, by measuring the longest diameter across the tumor and its corresponding perpendicular diameter (Length × Width^2^ × 0.52). Data was analyzed independently by the biostatistician.

### Illumina gene expression

Total RNA was extracted from Tet-inducible TUSC2 H1299 clone in treatments groups using RNeasy plus micro kits (Qiagen). Quality was assessed with a 2100 Bioanalyzer (Agilent). RNA (300 ng) was reversed transcribed to generate amplified biotinylated cRNA according to Eberwine procedure using Illumina TotalPrep RNA Amplification kit. cRNA (750 ng) was hybridized overnight to Illumina HT-12 Bead Arrays, washed and stained with streptavidin-Cy3 (Amersham-Pharmacia Biotech) according to the Illumina protocol. Arrays were scanned on a Bead Array Reader (Illumina). Raw measurements of the intensity of each bead were captured directly and processed as “bead-level”. All measurements were processed as “probe-level” data by GenomeStudio software (Illumina). One way ANOVA was used to identify the differential expressed genes among treatment groups. Beta-Uniform Mixture models were used to adjust for multiple comparisons. For the significant genes identified by One Way ANOVA, Tukey’s HSD Tests were used to do the post hoc analysis for pairwise comparisons. Each group had three replicates. Treatment groups were compared with controls and each binary combination was compared with AF alone. Finally, combinations of AF-TUSC2-erlotinib and TUSC2-erlotinib were compared to each other. Genes with p < 0.05 (by Tukey’s HSD tests) and fold change larger than 2 or smaller than −2 were considered as differentially expressed. Volcano plots and Heatmap for each comparison was generated using the R gplots program. Ingenuity pathway analysis based on statistical significance and strength of association with extracts was used for z-score.

### TXNRD1 assay

Thioredoxin Reductase 1 (TXNRD1) activity was measured using Thioredoxin Reductase 1 (TXNRD1) Activity Assay Kit according to the instructions (abcam, Cambridge MA). Briefly, the assay uses a 96-well plate with an antibody specific to TXNRD1 to isolate the enzyme pre-coated onto the wells. Following treatments, Tet-inducible TUSC2 H1299 cell lysates were prepared and incubated in the absence or presence of DTT (10 mM) at 37 °C in TE buffer for 15 min. Then, TXNRD1 was purified using Chroma Spin TE-10 columns to remove DTT. Aliquots of purified TXNRD1 (final concentration 1 μM) were incubated with increasing concentrations of NAPQI (0.1–100 μM) or DMSO in a final volume of 100 μl of TE buffer at room temperature. After 30 min, 100 μl of a TXNRD1/insulin mixture (50 nM purified rat liver TXNRD1, 0.5 mM NADPH, and 170 μM insulin in TE buffer) was added and changes in absorbance at 340 nm were recorded. TXNRD1 activity was calculated as the linear change in absorbance per min and expressed as a percentage of the enzyme activity of DMSO-treated control samples.

### ROS Intracellular Activity

Tet-inducible TUSC2 H1299 cells were cultured in a black 96-well culture plate for 24 hours and washed with HBSS before incubation in 100 μl of 1X cell-permeable fluorogenic probe 2’, 7’-Dichlorodihydrofluorescin diacetate (DCFH-DA) at 37 °C for 60 minutes. Cells were treated with 1 μg/ml doxycycline for 24 hours to induce TUSC2 and exposed to 0.7 μM auranofin and/or 0.9 μM erlotinib for two hours. All treatments were in triplicates. Fluorescence was analyzed with a fluorometric plate reader at 480/530 nm. Hydrogen peroxide, H_2_O_2,_ at 1000 μM was used a positive control.

### Statistical analysis

Statistical analysis was performed with SAS 9.4 (SAS Institute Inc., Cary, North Carolina, USA). All data are presented as mean  ± SD. The statistical significance of differences between treatments was tested by using three-way ANOVA and two-tailed t test; P < 0.05 was considered significant. The magnitude of drug interaction being synergistic, additive, or antagonistic was assessed by The Loewe additivity model^25^. CalcuSyn was used to generate median effect plot and 3D isobologram. S-plus 8.2 (TIBCO Software Inc., Palto Alto, CA) was used to calculate Interaction Index (II) and its 95% confidence interval. The cooperative effect of DC-TUSC2 and erlotinib and auranofin combination was assessed using a Bayesian parametric bootstrapping approach. The Statistical software S-PLUS 8.2 and SAS 9.4 were used for all analyses. Treatment effect in animal model was measured by the mean tumor volume on a log-scale in each treatment arm. The treatment effects of 4 groups were compared by ANOVA and pairwise mean comparisons were assessed with and without Turkey’s adjustment. Overall survival of animals with various treatments was estimated by Kaplan-Meier method. Log-rank (Mantel-Cox) test was performed to compare overall survival between different treatments.

## Additional Information

**How to cite this article**: Xiaobo, C. *et al*. TUSC2(FUS1)-erlotinib Induced Vulnerabilities in Epidermal Growth Factor Receptor(EGFR) Wildtype Non-small Cell Lung Cancer(NSCLC) Targeted by the Repurposed Drug Auranofin. *Sci. Rep.* **6**, 35741; doi: 10.1038/srep35741 (2016).

**Publisher’s note**: Springer Nature remains neutral with regard to jurisdictional claims in published maps and institutional affiliations.

## Supplementary Material

Supplementary Information
