# Supplementary material for: TUSC2(FUS1)-erlotinib Induced Vulnerabilities in Epidermal Growth Factor Receptor(EGFR) Wildtype Non-small Cell Lung Cancer(NSCLC) Targeted by the Repurposed Drug Auranofin
Source: Sci Rep. 2016 Nov 15;6:35741. doi: 10.1038/srep35741 (PMC5109231; doi:10.1038/srep35741)
Supplement: Supplementary Information [file srep35741-s1.pdf]

Supplementary information *for TUSC2(FUS1)-erlotinib Induced Vulnerabilities in Epidermal Growth Factor Receptor(EGFR) Wildtype Non-small Cell Lung Cancer(NSCLC) Targeted by the Repurposed Drug Auranofin, X. Cao, M. Majidi, M Feng, R Shao, J Wang, Y Zhao, V Baladandayuthapani, J Song, B Fang, L Ji, R Mehran, and J Roth*

Establish Tet-on inducible TUSC2 clones. Lung cancer cell lines H157 and H1299 with doxycycline dose response inducible TUSC2 expression were developed using Lenti-X Tet-On advanced inducible expression system (Clontech, Mountain View, CA). H1299, and H157 cells were infected with lentivirus generated with the pLVX-Tet-On advanced vector to constitutively express the tetracycline-controlled transactivator. After G418 selection, tetracycline-controlled transactivator positive colonies were further infected with pLVX-Tight-Puro-TUSC2 virus. 2nd generation colonies were selected and expanded under G418 and puromycin. Surviving clones were selected according to TUSC2 expression induction. The abilities of TUSC2 protein expression induction in H1299 and H157 Tet-on TUSC2 clones were revealed via western blotting, shown in supplementary figure 1. TUSC2 proteins significantly increased upon Doxycycline treatments whilst β-actin remains unchanged. The TUSC2 expression was positively correlated to Doxycycline doses.

Gene signature of auranofin treatment in H1299 Tet-on inducible TUSC2 clones. Total RNAs were extracted from Tet-inducible TUSC2 H1299 clones exposed to four different treatments, including control, TUSC2 induction via doxycycline, erlotinib and doxycycline-erlotinib. These RNAs were used as templates to generate amplified biotinylated cRNAs. These cRNAs (750 ng) were then hybridized overnight to Illumina HT-12 Bead Arrays, washed and stained with

streptavidin-Cy3 (Amersham-Pharmacia Biotech). Array signals were digitized using a Bead Array Reader (Illumina). The differentially expressed genes among four groups were revealed by Beta-Uniform Mixture models and one way ANOVA analysis. Tukey's HSD Tests were used to do the post hoc analysis for pairwise comparisons. Each group is the combination of triplicated samples. Genes with $p < 0.05$ (by Tukey's HSD tests) and fold change larger than 2 or smaller than -2 were considered as differentially expressed. Heatmap for the comparison was generated using the R gplots program, illustrated as supplementary figure 2a. List of top genes significantly down was shown in supplementary figure 2b. Cluster of oxidative stress related genes, such as HSPA6, IFNL2, PPP1R15A (GADD34), and GADD45B, were significantly downregulated after doxycycline-erlotinib treatment. Since elevated ROS level is lethal for the cancer cells, vulnerabilities in ROS mitigation created by doxycycline-erlotinib can be further exploited in combination with ROS boosters.

Supplementary Figure 1. H157 Tet-On TUSC2 and H1299 Tet-On TUSC2 cells were exposed to various doses of Doxycycline for 48 hours. Then proteins from these cells were subject to anti –TUSC2 western blotting. β-actin expression was also measured as loading control.

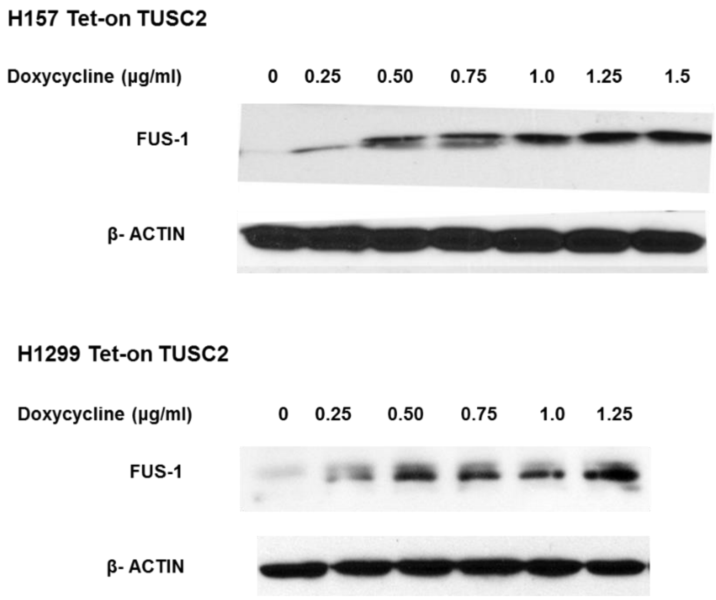

Supplementary Figure 2: Gene expression profile and pathway analysis. Illumina array Human HT-12V4 expression bead chip platform was used to compare the difference of gene expression profiles in four treatment groups, control, TUSC2 induction via doxycycline, erlotinib and doxycycline-erlotinib. Each treatment group was in triplicates. Beta-Uniform Mixture (BUM) models were used to adjust for multiple comparisons. Significant genes were identified by One Way ANOVA, and Tukey's HSD tests were used to analyze pairwise comparisons. Fold change larger than 2 or smaller than -2 were considered as significant at $p<0.05$. A) Heatmap of 887 significant genes with FDR of 0.0001. Control: gray, TUSC2 (doxycycline): blue, erlotinib: green, doxycycline-erlotinib: violet. b) List of top genes significantly downregulated. A fold change > 2 was considered significant.

A.

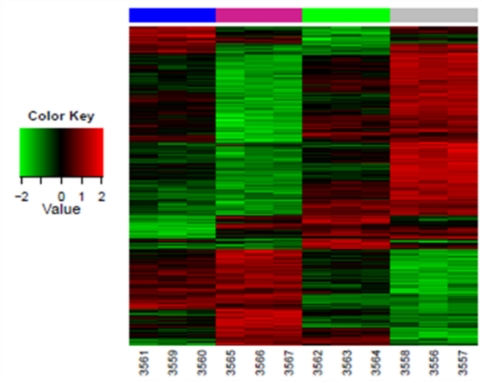

B.

Fold Change Down-Regulate

Molecules		Exp. Value
HSPA6	v	4.018
EGR1	v	3.694
IFNL2	v	3.415
IFIT2	v	3.136
IFNL1	v	2.496
PPP1R15A	v	1.996
TRA2B	v	1.978
GADD45B	v	1.914
PTGER4	v	1.899
WSB1	v	1.886
